# An open-source microscopy framework for simultaneous control of image acquisition, reconstruction, and analysis

**DOI:** 10.1016/j.ohx.2023.e00400

**Published:** 2023-02-06

**Authors:** Xavier Casas Moreno, Mariline Mendes Silva, Johannes Roos, Francesca Pennacchietti, Nils Norlin, Ilaria Testa

**Affiliations:** a Science for Life Laboratory, Department of Applied Physics, KTH Royal Institute of Technology, 171 65 Stockholm Sweden; b Interdisciplinary Institute for Neuroscience, CNRS UMR 5297, 33000 Bordeaux, France; c Department of Experimental Medical Science, Lund University Bioimaging Centre (LBIC), 221 00 Lund University, Sweden

**Keywords:** RESOLFT, Automation, Software

## Abstract

We present a computational framework to simultaneously perform image acquisition, reconstruction, and analysis in the context of open-source microscopy automation. The setup features multiple computer units intersecting software with hardware devices and achieves automation using python scripts. In practice, script files are executed in the acquisition computer and can perform any experiment by modifying the state of the hardware devices and accessing experimental data. The presented framework achieves concurrency by using multiple instances of ImSwitch and napari working simultaneously. ImSwitch is a flexible and modular open-source software package for microscope control, and napari is a multidimensional image viewer for scientific image analysis.

The presented framework implements a system based on file watching, where multiple units monitor a filesystem that acts as the synchronization primitive. The proposed solution is valid for any microscope setup, supporting various biological applications. The only necessary element is a shared filesystem, common in any standard laboratory, even in resource-constrained settings. The file watcher functionality in Python can be easily integrated into other python-based software.

We demonstrate the proposed solution by performing tiling experiments using the molecular nanoscale live imaging with sectioning ability (MoNaLISA) microscope, a high-throughput super-resolution microscope based on reversible saturable optical fluorescence transitions (RESOLFT).

## Graphical abstract

**Figure:**
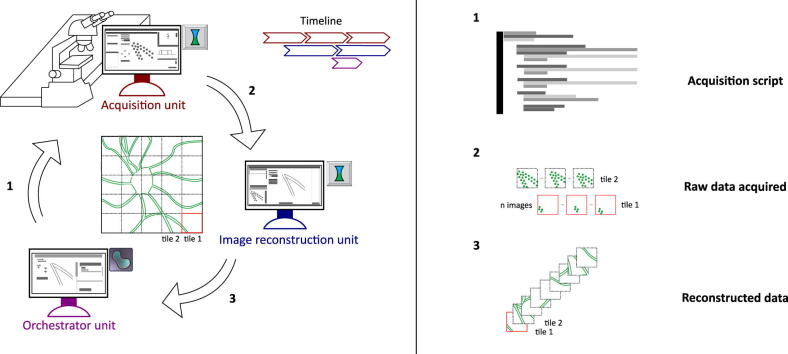

## Specifications table

**Table:** 

Hardware name	Sync-Scope
Subject area	Biological sciences
Hardware type	Imaging tools
Closest commercial analog	MetaMorph, Molecular Devices
Open source license	GNU General Public License (GPL)
Cost of hardware	2.000–4.000 EUR
Source file repository	https://zenodo.org/record/7561142

## Hardware in context

Microscopy is a powerful tool for cell biology. Still, it becomes costly and time-consuming when pushed towards high throughput, such as when imaging extended areas for a prolonged time. Therefore, microscopy automation is an increasing area of interest in biological imaging [1] and high-throughput screening [2], [3] applications. However, automation imposes multiple challenges from the hardware and software integration side, particularly regarding computational efficiency and workload distribution. Most solutions often rely on commercial software and hardware, making it difficult to adapt to different microscopy modalities and extend support to new applications.

Open-source software and hardware solutions for microscopy automation are relatively new, and most either rely on commercial microscopes [4], [5] or are tailored to a specific technique [6], [7], [8], [9], [10]. Different general solutions have been proposed, such as Pycromanager [11], AutoScanJ [12], and MicroMator [13]. They interface with µManager [14], a well-established open-source software package with extensive driver support, distributed as an ImageJ [15] plugin. However, µManager presents limitations as the complexity of the microscopy modalities increases. Some examples are controlling multiple cameras independently, synchronizing multiple hardware devices, performing simultaneous image acquisition and reconstruction, or controlling specialized devices such as spatial light modulators or point detectors. Python-based software alternatives have been developed [16], [17], [18], [19], making debugging and contributing more convenient in contrast to Java.

Sample-adaptive automation methods for image acquisition have been recently proposed [20], [21], in which experiments and image analysis are combined to achieve fast, directed imaging by adapting to events in the sample of interest. Furthermore, microscopy automation in high-throughput imaging has been implemented using a custom-made low-cost microscope [22]. Imaging extended sample areas by tiling has been previously achieved through microscope automation in light-sheet [23], [24] and STED [25] microscopy.

Several microscopy modalities require a reconstruction algorithm to turn the acquired data into final images. Techniques such as single-molecule localization microscopy (SMLM) [26], [27], [28], parallelized RESOLFT [29], [30], [31], [32], structured illumination microscopy (SIM) [33], [34], super-resolution optical fluctuation imaging (SOFI) [35], universal live-cell super-resolution microscopy (SRRF) [36], among others, require reconstructions algorithms to output a high-resolution image from the experimentally-acquired series of raw images. Furthermore, light field microscopy (LFM) [37], Fourier ptychography microscopy (FPM) [38], and lens-free on-chip microscopy [39] require computational algorithms to generate microscopy images by gathering information from different sources, such as the angle of the illumination light. However, the reconstruction procedure is often performed sequentially after all image data is acquired. This can be a limiting factor if the users need a rapid answer to adapt imaging schemes, optimize sample preparation or perform time-consuming experiments. Multiple efforts have been placed to accelerate the reconstruction process using a Graphics Processing Unit (GPU) [40], [41], [42], [43]. Nevertheless, these techniques would greatly benefit from distributing computational resources and scheduling the experimental tasks in real time.

Image analysis of data in real-time is a promising direction, with solutions like ImJoy [44], BioImageIT [45], and cell profiler [46]. They implement several algorithms and plugins, which can, in principle, be applied to data acquired directly from the microscope. However, combining these pipelines with the entire experimental acquisition scheme, for example, using their output as active feedback on the acquisition, reminds a challenge. Therefore, a general open-source framework that integrates image acquisition, reconstruction, visualization, and data analysis and flexibly adapts to different microscopy modalities is still missing.

Here we present a framework to simultaneously perform image acquisition, reconstruction, and analysis using ImSwitch [19] and napari [47]. On the one hand, ImSwitch is an open-source software solution for controlling microscopes, adaptable to different levels of complexity. It includes image acquisition and reconstruction modules, where the experimental images can be visualized in real-time. On the other hand, napari is a community-driven image viewer with multiple plugins for image analysis and is widely used in the microscopy field. Our solution is based on the simple concept of file watching, where different units monitor a filesystem searching for new files to process and add the arriving items into a queue.

The solution doesn't require implementing any client–server-based approach, which relieves the burden of using a centralized server in terms of complexity, security, and dependency on other software packages. We provide a scripting engine in python that enables microscopy automation of any experiment designed by the user. The experiments are orchestrated remotely by creating and editing the scripts and adding them to the acquisition unit queue. However, the list of microscope commands is executed directly in the acquisition unit instead of running independent hardware orders through function calls over a network, which would limit the experiment's time resolution. Security is not a concern since the computer units are assumed to be in the same laboratory or institute, or remotely shared with specific and known users. The proposed framework can even be implemented without an internet connection, either by using multiple computers or running all the instances on the same machine, just by having a local or remote filesystem.

We provide the design files containing the main functionality of the file watcher as well as its integration with napari and ImSwitch. Furthermore, it can be easily implemented in other python-based software [16], [17], [18] both for acquisition and reconstruction. For example, the user can develop their own python scripts for image reconstruction and add them to the workflow by including the file watcher model.

The imaging data and metadata are fetched from ImSwitch and saved into either Zarr or HDF5 files, and the reconstructed images are then saved in both OME-Zarr and Tiff. OME-Zarr is an implementation of the Zarr format using the Open Microscopy Environment (OME) specifications [48]. Zarr can store chunks of data in a directory tree, which is highly beneficial for access times in a shared filesystem. The chunk retrieval time was compared to HDF5 and Tiff, and proven to be less sensitive to data location. This is the main reason we used Zarr for our experiments when using different physical computers. We have implemented a napari plugin that orchestrates the ImSwitch experiments and displays the images as layers. The plugin displays the metadata of each file to enable experimental reproducibility.

Increasing the microscope throughput is a common challenge in super-resolution microscopy. In SMLM, studies have shown how illuminating the sample with uniform illumination [49], [50] increases the field of view up to 200 × 200 µm^2^. Using parallelized illumination and specialized optics [30], [51] in RESOLFT, up to 130 × 130 µm^2^ was reached. These approaches can be combined with tiling to extend the throughput further. We apply the proposed framework to tiling super-resolution images using the molecular nanoscale live imaging with sectioning ability (MoNaLISA) [31], a microscope based on the concept of reversible saturable optical fluorescence transitions (RESOLFT) [52], [53], [54]. The microscope setup aims at parallelizing the illumination using patterned light to extend the field of view (FOV) and achieve faster recordings. Our approach provides a general framework that can be applied to a variety of microscopy techniques and experiments to increase the throughput not only in acquisition but also in reconstruction and analysis.

We have focused on parallelizing the acquisition and reconstruction tasks by distributing the computational workload in different units, further increasing the throughput of the technique. We have performed two experiments that encompass tiling and timelapse imaging to extend the microscope throughput further. We imaged actin cytoskeleton and mitochondria in human epithelial cells and other cells of increased morphological complexity, such as neurons. The proposed solution extends the recording to multiple cells and can be applied to whole sample screening in theory. As proof of principle, we recorded a FOV of 160x160µm^2^ from 5x5 tiles of 38x38µm^2^. The experiment was performed using a Python script executed in ImSwitch, which follows two steps: (1) widefield-based user registration of the focus in each tile, and (2) automatic RESOLFT imaging. The registration was needed to compensate for movement and tilt of the sample respect to the stage. We added overlap between the tiles so that the reconstructed images could be aligned in a post-processing step, reducing the constrain of stage precision in (x, y).

## Hardware description

**Fig. 1:**
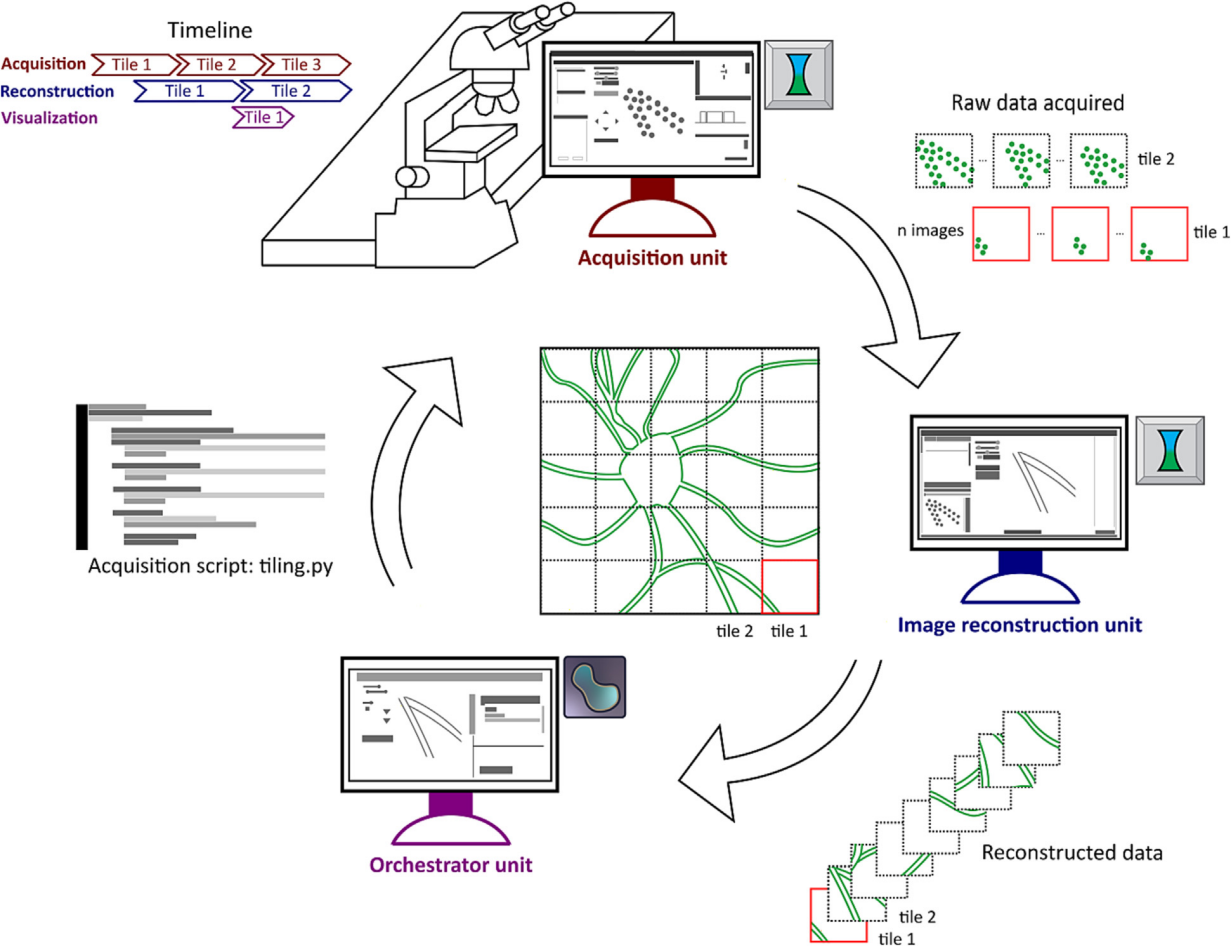
General framework for simultaneous experiment acquisition, image reconstruction, and visualization using multiple computational units. The microscope experiments are performed in the acquisition unit by executing python scripts, which are created and distributed by the orchestrator unit. The user specifies the acquisition parameters in the scripts, such as number of tiles and laser powers. The recorded data is saved on disk (raw data), which acts as a queue for the image reconstruction unit. The image reconstruction unit turns the acquired raw data into tiles in a parallelized manner (see timeline in the top left corner) and can be visualized and post-processed in the orchestrator unit. Repeating this cycle gives an image of a neuron with an increased FOV.

We present a file-based synchronization framework to simultaneously perform image acquisition, reconstruction, and analysis in microscopy applications. Fig. 1 exemplifies a typical microscope automation procedure using the proposed framework, where three units are synchronized to perform high-throughput imaging of a neuronal cell by tiling. One cycle consists of (1) imaging a FOV (i.e., tile) using a microscope and the acquisition unit, which will generate the raw data; (2) reconstructing the raw data into a final image of the tile; and (3) visualizing the image as a napari layer and post-processing. The cycle is concurrently performed multiple times. Essentially, the units will run independently (see Fig. 1 timeline) to extend the FOV by repeating the cycle for each tile. All the tiles will then be stitched to build the final image, thus expanding the microscope's throughput. The execution is handled using python scripts, which contain all the commands to perform the experiments, acquire data in each tile, move the stage between each tile, and collect the user parameters from the GUI.

It is important to remark that the presented approach is not limited to a specific number of units, and multiple units can run on the same physical computer. Each implementation will depend on two factors: the performance and specifications of the computers and the requirements of the experimental acquisition and data reconstruction applications (CPU usage, RAM occupied, and time of processing). For example, in microscopy applications where the recorded images are large, the reconstruction process might allocate a significant portion of the RAM and result in computer freezing, compromising the experiments. In this case, using separate computers for each unit is beneficial to maximize the resources. Then, the acquisition will not be affected by delays in the reconstruction unit.

If the resources of the computer allow, the framework can run on the same computer. This can be implemented either with the same unit for acquisition, reconstruction, and analysis using multithreading or by distributing the three tasks to different units. We chose the latter approach because it offers two benefits with respect to the first. Firstly, it is compatible with multiple software for data acquisition and image reconstruction. For example, the framework can use ImSwitch for acquisition and a Python script for reconstruction using the File Watcher python file provided (Design Files). Secondly, multiple microscopes (acquisition units) can delegate to the same unit for image reconstruction, which can be beneficial in microscope facilities and laboratories.

Each of the presented units serves a different task: acquisition, reconstruction, and orchestration. Firstly, the acquisition unit features an ImSwitch instance with the control module – *imcontrol*. This unit will perform experiments on the microscope by controlling hardware devices and storing the imaging data (raw data) and experimental metadata in disk or RAM. Each experiment is represented by a python script file and is executed in the scripting module of ImSwitch – *imscripting*. The scripts have access to the custom-designed and exported Application Programming Interface (API), which can be easily extended to include new functionality. In practice, scripts can automate the microscope by changing the state of any hardware device and accessing the resulting data. Secondly, the reconstruction unit features an ImSwitch instance with the reconstruction module – *imreconstruct.* This module is designed for those microscopy applications that require signal-processing algorithms to generate the final images from the raw data (e.g., n frames are reconstructed into one image). Finally, the orchestrator instance features a napari instance with the *napari-file-watcher* plugin. The plugin provides basic script editing functionality and displays the new reconstructed images as napari layers for further analysis. The user performs experiments using the script editing module in the orchestrator graphical user interface (GUI) and visualizes the incoming data as image layers.

The benefit of using napari for image analysis is that the reconstructed data can be post-processed by one of the available plugins in the napari hub (for example, image segmentation). Users can also develop plugins and contribute to the broader community. Image reconstruction is separated from visualization to cover those applications where raw data require specialized scripts for reconstruction that are not distributed as napari plugins.

The proposed solution can be extended to other experimental applications by simply designing different scripts, for example, performing a 3D stack or timelapse imaging. A complete list of API functions for scripting is available in the *readthedocs* documentation of ImSwitch, as well as instructions on adding new functionality (https://imswitch.readthedocs.io/en/stable/). It also contains a detailed description of the experimental metadata of ImSwitch and how to load it, the GUI components, and information on how to expand ImSwitch to multiple microscopy modalities and hardware devices. Furthermore, the framework can be generalized to other low-cost computing devices, such as Raspberry Pi, Arduino, and Jetson Nano. The only requirement is a shared file system and a python software package for image reconstruction and file watching.

**Fig. 2:**
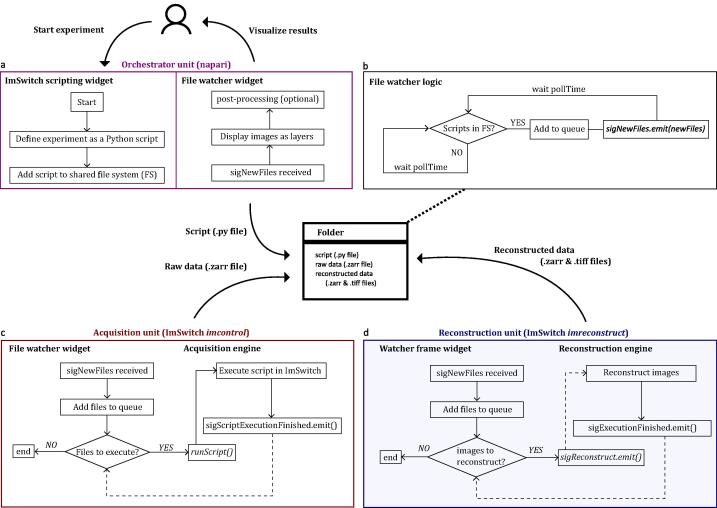
Software pipeline using multiple units. a. The orchestrator is an instance of napari with the *napari-file-watcher* plugin*.* The user interacts with the orchestrator unit by defining experiments as python scripts, which are then added to the filesystem using the ImSwitch scripting widget. Once the reconstructed images are received, they are displayed as napari layers available for post-processing. b. The logic for each file watcher in Python. It periodically monitors a filesystem (FS), adds the incoming files to a queue and sends them using the *sigNewFiles* signal. c. The acquisition unit is an instance of *ImSwitch.* The file watcher widget displays and tracks the new files in each folder. Whenever new files are in the FS, it adds them to a queue and executes them sequentially. After every execution, the signal *sigScriptExecutionFinished* is called. d. The reconstruction unit is also an instance of ImSwitch, featuring the *imreconstruct* module. The workflow is similar to the acquisition, with the difference that the files are raw images that are reconstructed with an image processing algorithm. The reconstruction function is not called directly but through the *sigReconstruct* signal instead. This choice is due to the architecture of the *imreconstruct* module. Solid lines represent direct connections, and dotted lines are calls using python signals.

The software pipeline is described in Fig. 2. The user interacts with the orchestrator unit, which is implemented as a napari plugin (*napari-file-watcher*) and contains two widgets (Fig. 2a). The *ImSwitch scripting* widget implements an editor that the user can use to define experiments and adjust imaging parameters. Once the script is finalized, it is added to the shared filesystem (FS). The second widget, the *File watcher,* displays the experimental results as napari layers (already reconstructed). The user can post-process the images using existing napari plugins or develop other processing pipelines on top.

Each unit implements the file watcher thread (Fig. 2b) to monitor a folder periodically (pollTime) until new files arrive. Then, it adds the files to a queue and sends them using the signal *sigNewFiles.* This signal is connected to the main engine in each unit so that files are processed. We provide the file for the file watcher thread so that it can be used by other python software in any of the units.

The acquisition and reconstruction units are similar (Fig. 2c-d). Each of them implements a file watcher thread that monitors a folder selected by the user and then adds the files to a queue. Each file is processed sequentially, and a signal is emitted when each execution is finalized. The acquisition unit deals with python scripts that execute the experiments (*runScript*) and emits the signal *sigScriptExecutionFinished.* The reconstruction handles raw images that are reconstructed, followed by emitting the signal *sigExecutionFinished*.

The user can select between saving the raw images in Zarr or HDF5 files in the acquisition unit. The metadata is also included as an attribute (*ImSwitchData)* and contains all the experimental details needed for reproducibility. The reconstruction unit saves the data in both TIFF and Zarr files.

The file watcher functionality is implemented in the model layer of ImSwitch, and the orchestrator as an independent plugin distributed in the napari hub. The widgets can be easily applied to other python software packages and monitor any file extension added to the initialization function, providing generalization to multiple applications. A logger file is written into the folder once the experiment is finalized. It contains information about the computer name and starting date, all the files processed, and the time between arrival and end of execution of each item. Therefore, the performance of each computer can be easily evaluated for different reconstruction algorithms and experimental applications using resource monitor software, such as the Resource Manager in Windows.

## Design files summary

**Table:** 

Design file name	File type	Open source license	Location of the file
Computer framework: Fig. 1.eps	Figure	Creative Commons Attribution 4.0 International	Zenodo: https://zenodo.org/record/7561142
Automation pipeline: Fig. 2.eps	Figure	Creative Commons Attribution 4.0 International	Zenodo
Timelapse tiling script: tiling.py	Python script	Creative Commons Attribution 4.0 International	Zenodo
Selective tiling script incl. registration: timelapse.py	Python script	Creative Commons Attribution 4.0 International	Zenodo
Napari-file-watcher plugin: napari-file-watcher.zip	Python software	GNU General Public License (GPL)	Zenodo and https://www.napari-hub.org/plugins/napari-file-watcher release v0.1.1
File watcher model: FileWatcher.py	Python software	GNU General Public License (GPL)	Zenodo
File watcher ImSwitch acquisition widget: WatcherWidget (ImSwitch imcontrol).zip	Python software	GNU General Public License (GPL)	Zenodo
File watcher ImSwitch reconstruction widget: WatcherFrame (ImSwitch imreconstruct).zip	Python software	GNU General Public License (GPL)	Zenodo
Tiling widget: TilingWidget (ImSwitch imcontrol).zip	Python software	GNU General Public License (GPL)	Zenodo
ImSwitch version (ImSwitch-2.0.0.zip)	Python software	GNU General Public License (GPL)	Zenodo and https://github.com/kasasxav/ImSwitch release v2.0.0

## Bill of materials summary

**Table:** 

Designator	Component	Number	Cost per unit - currency	Total cost - currency	Source of materials	Material type
Acquisition unit	Dell Precision 5820 MT	1	2.500€	2.500€	Dell	
Reconstruction unit	HP Workstation Z2 Mini G3	1	1.500€	1.500€	Hewlett-Packard	

## Build instructions

The requirements to implement the framework using different computers are a microscope with a workstation (acquisition) and a second computer to perform the reconstructions in real-time. If the acquisition computer is powerful enough, both units can be executed in the same machine as well. The orchestrator can often be included in the acquisition or reconstruction computers because it requires a minimum workload. The user can implement real-time image analysis pipelines on top of the visualized images using napari plugins in the orchestrator unit.

ImSwitch must be installed in both the acquisition and reconstruction units and napari with the *napari-file-watcher* plugin in the orchestrator unit. This section provides a detailed description of the installation procedure and how to set up the framework for experimental orchestration. We recommend that the user or developer reads the ImSwitch online documentation to check for device compatibility.

### ImSwitch installation and configuration

We have released a new version of ImSwitch (v2.0.0) that supports the features presented in this article, which is included in the Python Package Index (PyPI). Therefore, the only necessary steps in order to install ImSwitch are to install conda and run the following commands:

*conda create -n imswitch-env*
*conda activate imswitch-env*
*pip install imswitch*
*imswitch*

After installing ImSwitch, a configuration folder is generated in “*Documents*” > “*ImSwitchConfig*”. A standard procedure is to add the setup file containing the hardware device list and software widgets to display in “*imcontrol_setups*” folder (see more in the documentation). Upon installation, there is already a list of example files, and upon initialization, the user can choose one of the available options. After ImSwitch is initialized, the setup file to load can be changed in “*Tools*” > “*Pick hardware setup*…”.

**Fig. 3:**
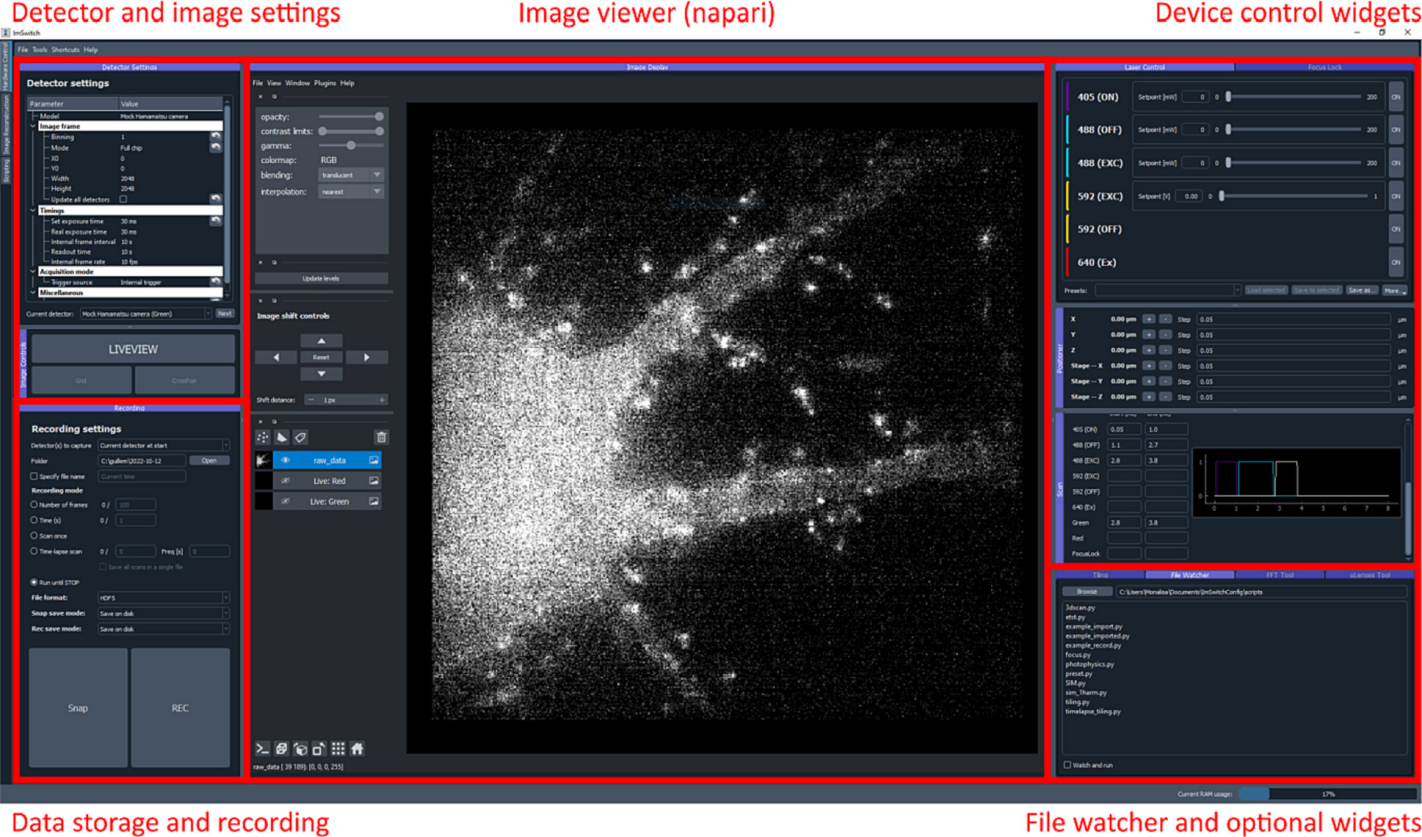
ImSwitch GUI, acquisition unit. The GUI implements different widgets for the control of devices (“*Device control widgets*”) and experimental acquisition (“*Detector and image settings*”). The “*Data storage and recording*” module saves data into disk or memory. The file watcher and other specialized widgets are implemented in “*File watcher and optional widgets*”.

The widget with the functionality for the file watcher needs to be included in the “*availableWidgets*” by the end of the setup file as “*Watcher*”. Fig. 3 shows the ImSwitch GUI in the acquisition unit for the microscope that we employed in the experimental part of this work (see Validation and Characterization).

The file “*config*”> “*modules*” contains a list of the GUI modules to be displayed. We recommend only having “*imcontrol*” and “*imscripting*” in the acquisition unit and “*imreconstruct*” in the reconstruction unit.

**Fig. 4:**
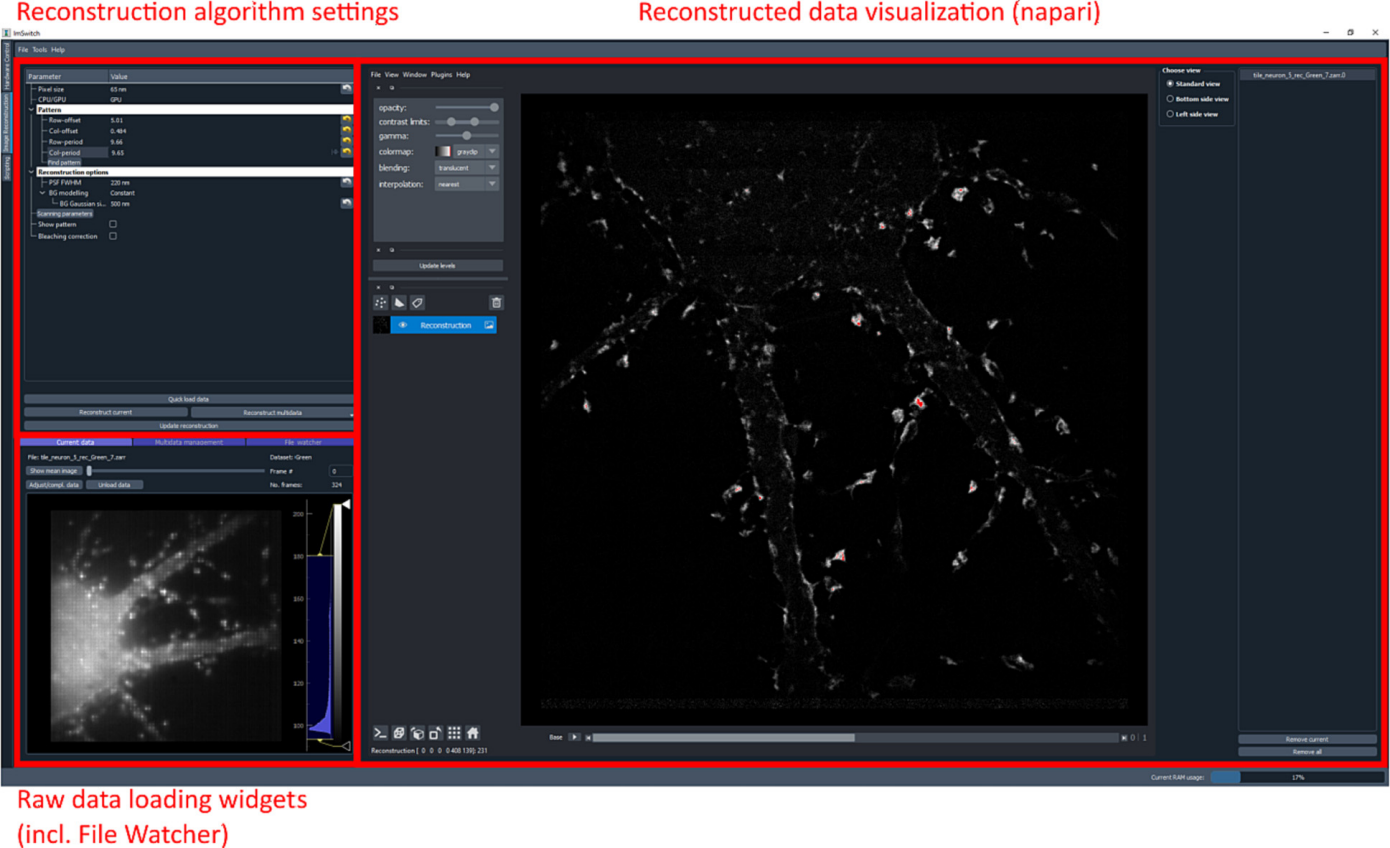
ImSwitch GUI, reconstruction unit. The raw data is displayed in the “*Raw data loading widgets*” section, and the reconstructed data is in the “*Reconstructed data visualization*” module. The reconstruction algorithm parameters can be set in “*Reconstruction algorithm settings*”.

Fig. 4 shows the reconstruction unit ImSwitch GUI. The reconstruction module is implemented for parallelized RESOLFT strategies. However, different modules can be implemented and added to ImSwitch. It is also possible to use other software reconstruction alternatives and implement the File Watcher widget, similar to how it is developed in the napari plugin.

### Napari installation

Napari is available in PyPI as well. Therefore, to install napari:

*conda create -n napari-env*
*conda activate napari-env*
*pip install napari*
*napari*

**Fig. 5:**
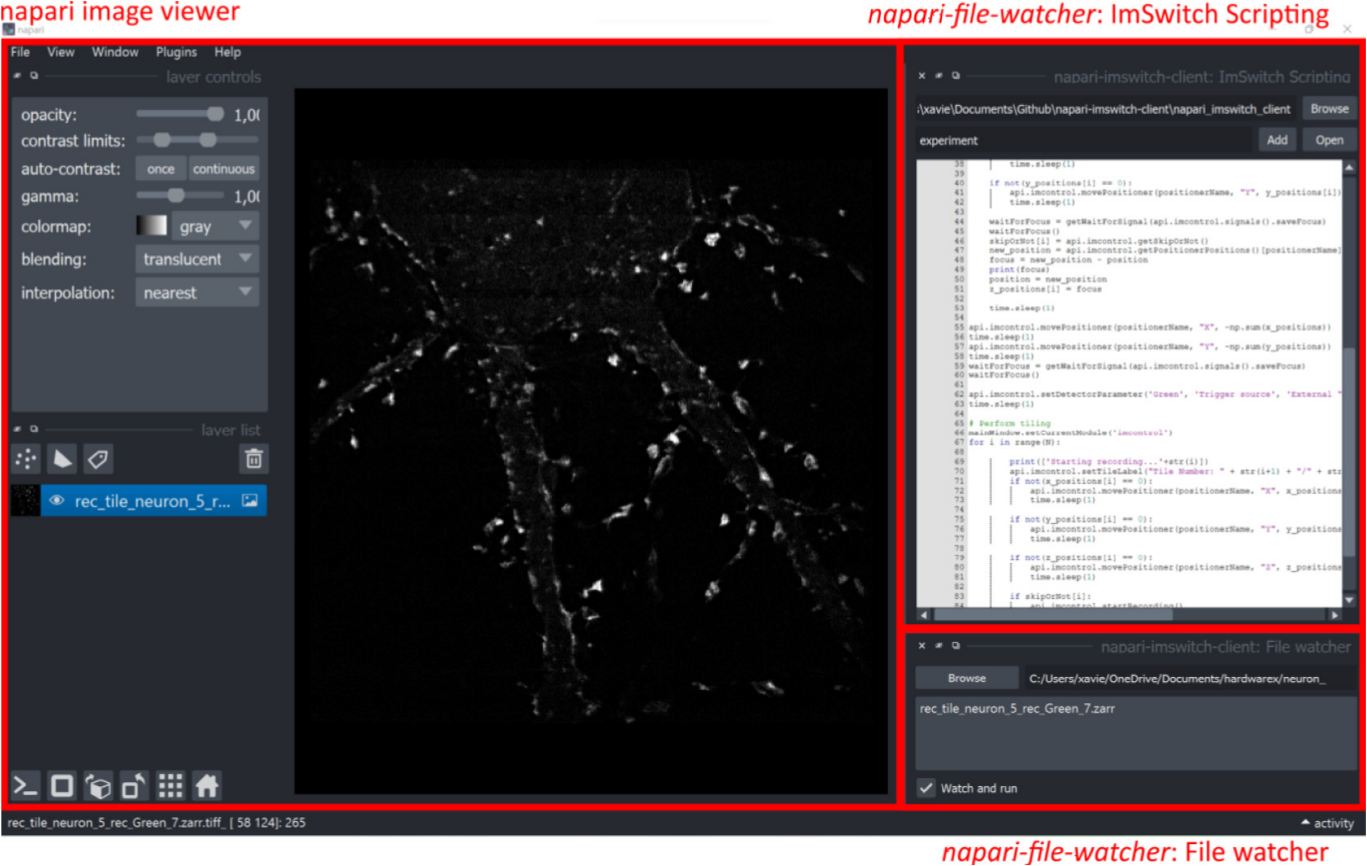
Napari GUI and the *napari-file-watcher* plugin, orchestrator unit. The plugin contains two widgets: “ImSwitch Scripting” for editing and creating execution scripts and the “File Watcher” waits for new images to be displayed.

After napari is installed, the plugin “*napari-file-watcher*” needs to be installed. The plugin can be installed from “*Plugins*”> “*Install/Uninstall Plugins…”*. The GUI is shown in Fig. 5.

## Operation instructions

Before starting the experiments, all units must be synchronized by setting up the file watchers. The user selects the filesystem folder, and folders for scripting and reconstruction are then automatically created. The following steps should be followed to set up the experimental framework:

1. In the acquisition unit, the scripting folder can be selected by clicking on the “*Browse*” button and then check the “*Watch and run*” box.
2. The best practice is to have a disk in the acquisition unit that can be shared over the network. This can be easily done in Windows by opening File Explorer and right-clicking on the disk of interest, and then “*Properties*” > “*Sharing*” > “*Advanced Sharing*” > “*Permissions*”. The user used in the reconstruction unit should be added here. However, other shared storage options are also possible, as long as both the acquisition and reconstruction units have writing access to the filesystem.
3. In the acquisition unit, in the recording settings widget (bottom left of the GUI), the user can select the folder where the experimental data will be saved, which will be a folder in the shared disk. Also, select Zarr as the file extension.
4. In the reconstruction unit, in the bottom-left corner and the widget “*File Watcher*,” the data folder should be selected, and then check the “*Watch and run*” box.
5. The reconstructed data will be saved in a folder named “*rec*” inside the data folder. Select that folder in the file watcher widget of the “*napari-file-watcher”* plugin, and then press “*Watch and run*.”
6. Finally, select the scripting folder (same as in 1.) from the scripting widget in the “*napari-file-watcher*” plugin, write scripts or open existing ones, and then start the experiment by pressing “*Add*”.

**Fig. 6:**
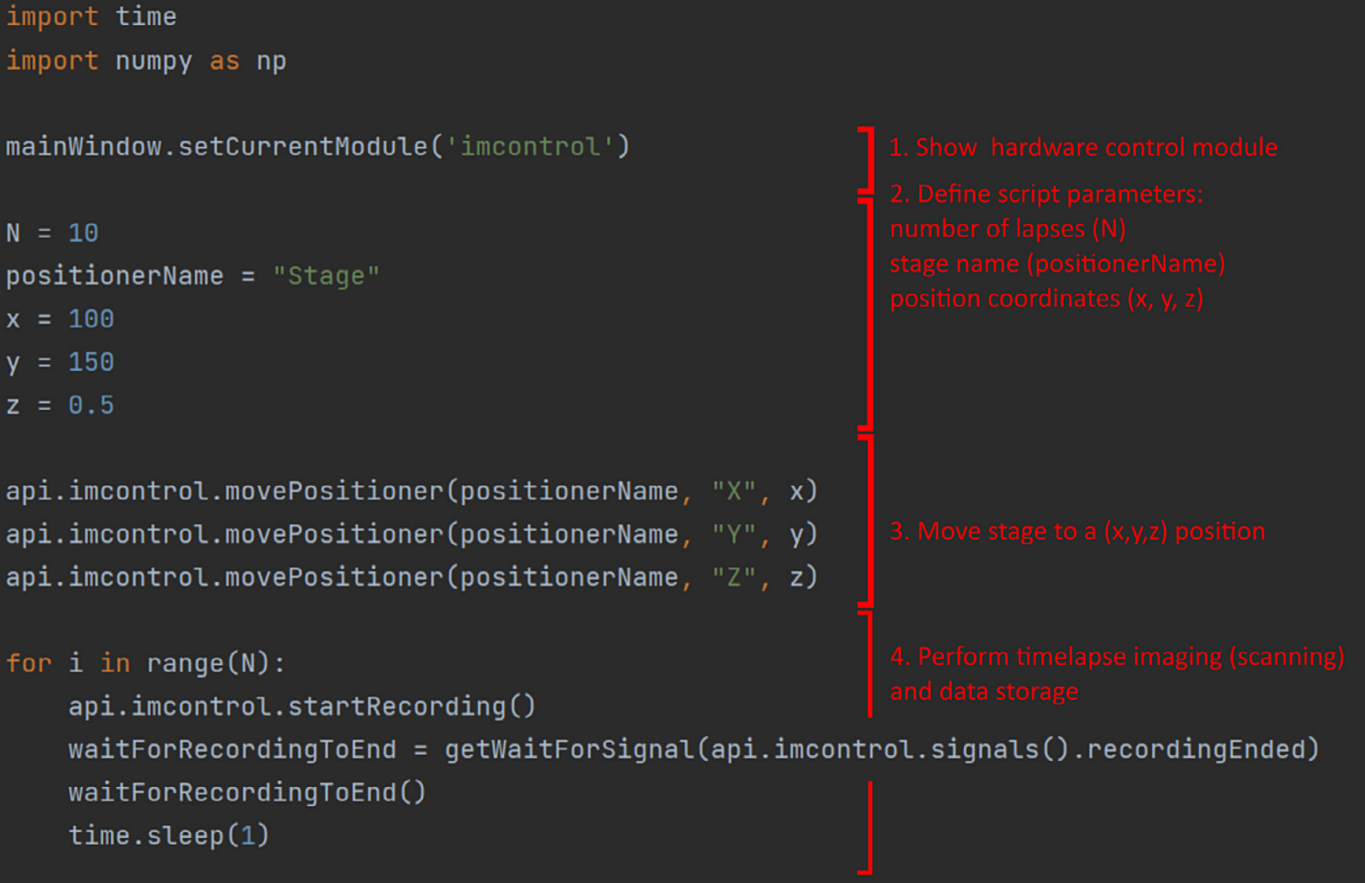
Timelapse ImSwitch automation script. N = 10 lapses are executed in the (x,y,z) stage position coordinates. The ImSwitch API is exported and accessible with the “*api.imcontrol*” call.

The script can access any API exported function, and new functions can be easily added using the @APIExport decorator in any controller. A list of the accessible functions is shown on the documentation page. Fig. 6 shows an example script for performing timelapse imaging of N = 10 lapses in the (x,y,z) coordinates. Each of the lapses is a scan-based experiment that saves the raw data on disk.

**Fig. 7:**
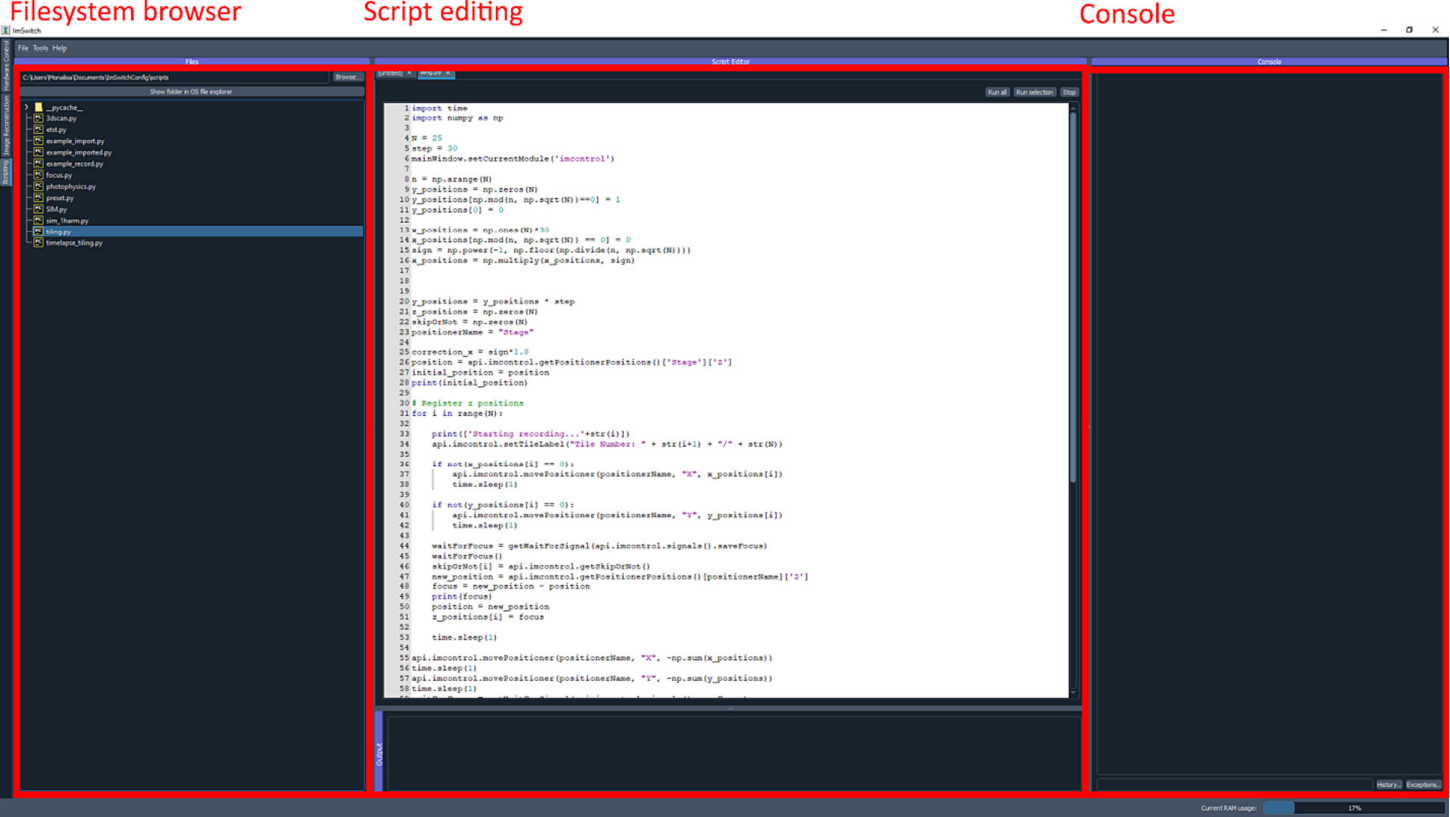
ImSwitch GUI in acquisition unit, *imscripting* module for editing and executing scripts. The scripts can be loaded in “*Filesystem browser*”, edited, and executed in the “*Script editing*” section, and a “*Console*” is implemented for debugging purposes.

The scripting engine is in charge of executing the files in the acquisition unit (*imscripting*). It also has a GUI, which we display in Fig. 7. It provides basic script editing functionality and the possibility to run and stop experiments. The GUI can be helpful first to test, debug and implement the experimental scripts locally before using the full framework. After the scripts are implemented, the orchestrator unit will be used to adapt the scripts further and send them to the acquisition unit.

## Validation and characterization

In order to validate and characterize the proposed framework, we have used the MoNaLISA [31] microscope. MoNaLISA is a parallelized RESOLFT method that offers a large FOV (up to 50x50µm^2^) with an increased speed (0.3–1.3 Hz). We chose two use cases related to timelapse and tiling images of extended regions for different biological structures: mitochondria and actin in U2OS and neuronal cells. The main focus is to automate the acquisition using scripts and increase the microscope throughput by distributing the acquisition and reconstruction workload using different devices.

### Observing mitochondrial dynamics with super-resolution microscopy

MoNaLISA has been previously employed to study mitochondria dynamics in U2OS cells [31], [32], [55]. Mitochondria is a highly dynamic organelle and demands an equally fast imaging strategy to follow its movements over the cell. Therefore, throughput in this biological application means (1) the possibility of acquiring multiple frames with a minimum delay caused by the computational acquisition and reconstruction and (2) observing the dynamics in multiple cells to acquire statistical relevance. Previously, to keep the temporal resolution, experiments were first performed, and the data were reconstructed and analyzed offline. However, there will be a delay until the user receives substantial feedback, such as whether the selected cells presented relevant dynamics. Visualization of experimental data in real-time is also crucial in the case of drug treatment when the administration of a drug needs to happen at a specific moment during the acquisition. Our approach can be beneficial to perform adaptive imaging, adjusting imaging parameters based on the analysis of the data [21].

The use case presented involves observing cell dynamics with super-resolution details, such as visualizing the outer membrane compartment in an extended region by performing a cyclic time-lapse experiment. In particular, extending the FOV can be obtained using a motorized stage that moves from tile to tile performing one acquisition at each position. If this procedure is repeated (here ten times), the cell dynamics can be observed in the entire extended area. In order to maintain the stability of the focus during the movement, a focus lock strategy is implemented following previously-published instructions [25]. The focus lock keeps the focus automatically over time by using the cover glass reflection and successfully adjusts to the 2x2 tiling movement.

**Fig. 8:**
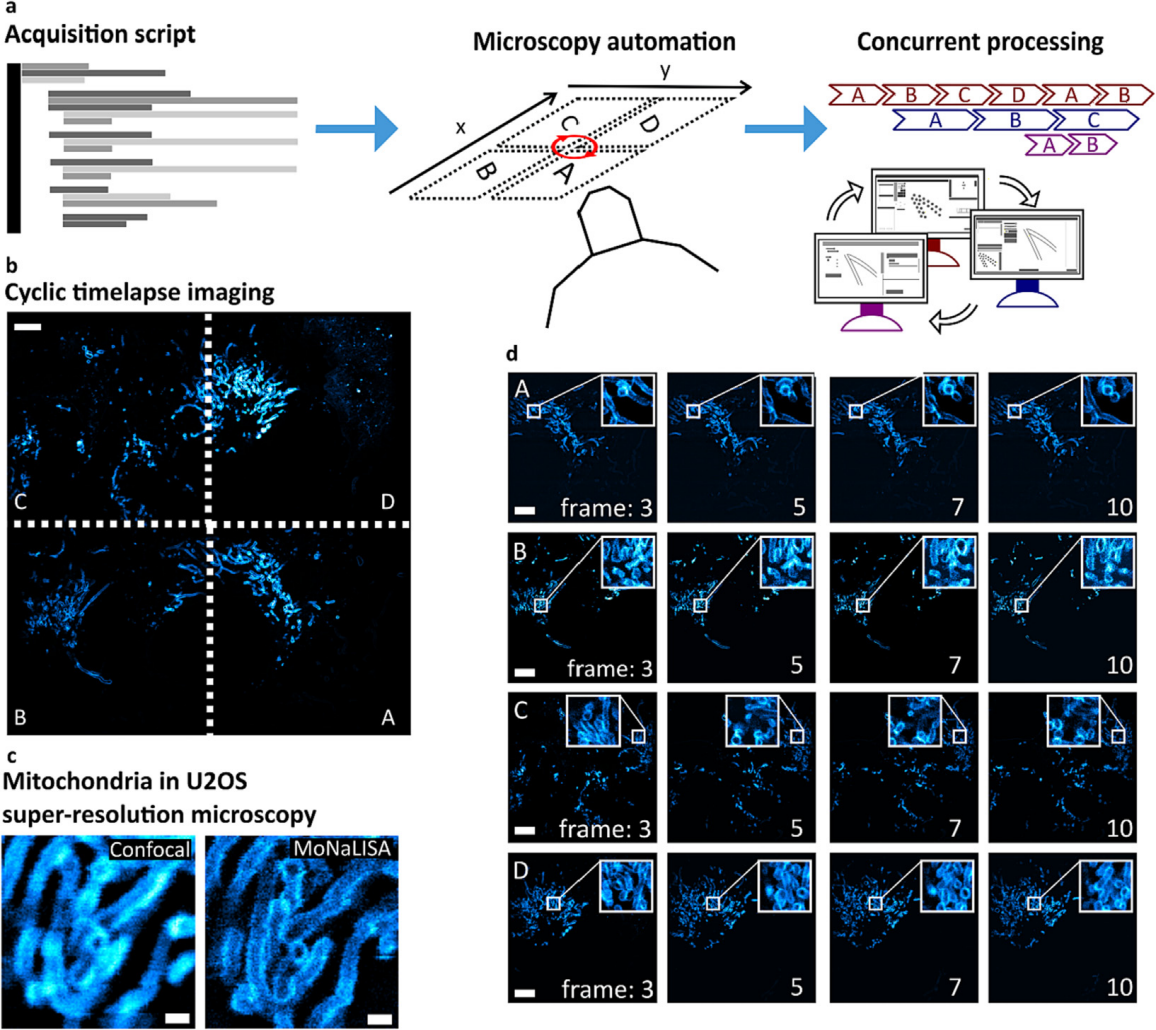
Cyclic time-lapse imaging of mitochondria in U2OS cells in a 2x2 (A, B, C, D) tile array. **a** The acquisition script (timelapse-tiling.py) drives the microscopy automation acquisition by calling ImSwitch functions. Concurrent processing enables subsequent acquisition and reconstruction of the image tiles. **b** Cyclic timelapse images are performed by imaging each of the tiles and sequentially repeating the procedure. Scale bar 5 µm. **c-d** The super-resolution features can be observed during a prolonged time (10 super-resolved frames) for each tile independently in U2OS cells expressing OMP25-rsEGFP2. A zoom-in visualizes the dynamics of selected areas in both confocal and MoNaLISA modalities. Scale bars 0.5 µm and 5 µm, respectively.

The experimental design and results are shown in Fig. 8. A script contains the experimental instructions to be sent to the microscope. In this case, we perform a MoNaLISA scan in every tile and save the files on the hard drive. In between scans, the motorized stage moves to the following area. We use the digital outputs of a data analog card (*NIDAQ PCI 6371*) to synchronize hardware devices (e.g., lasers and stage axis) with the camera (externally triggered) using a pulse scheme optimized for RESOLFT imaging. The script can successfully wait for the end of the scan by using exported signals in the *imscripting* module of ImSwitch.

By combining MoNaLISA with the presented multiunit framework, higher throughput can be achieved by distributing the reconstruction workload (limited mainly by the RAM usage) and improving the computation time while keeping the lateral and temporal resolution introduced by the technique.

### Selective tiling in neurons and U2OS cells with a user-driven approach

As a second application, we extended the FOV of the MoNaLISA microscope by applying the proposed framework in tiling experiments to achieve higher throughput. We imaged extended sample regions of neurons and U2OS cells. Neurons are highly polarized cells that communicate with each other via specialized sites called synapses, which occur along neuritic processes such as axons and dendrites. These processes grow from the cell body and expand over large areas with high morphological and functional complexity relevant to synaptic organization and transmission [51]. Therefore, tiling in super-resolution microscopy is a method that can highly benefit neuronal imaging by massively expanding the FOV [25].

**Fig. 9:**
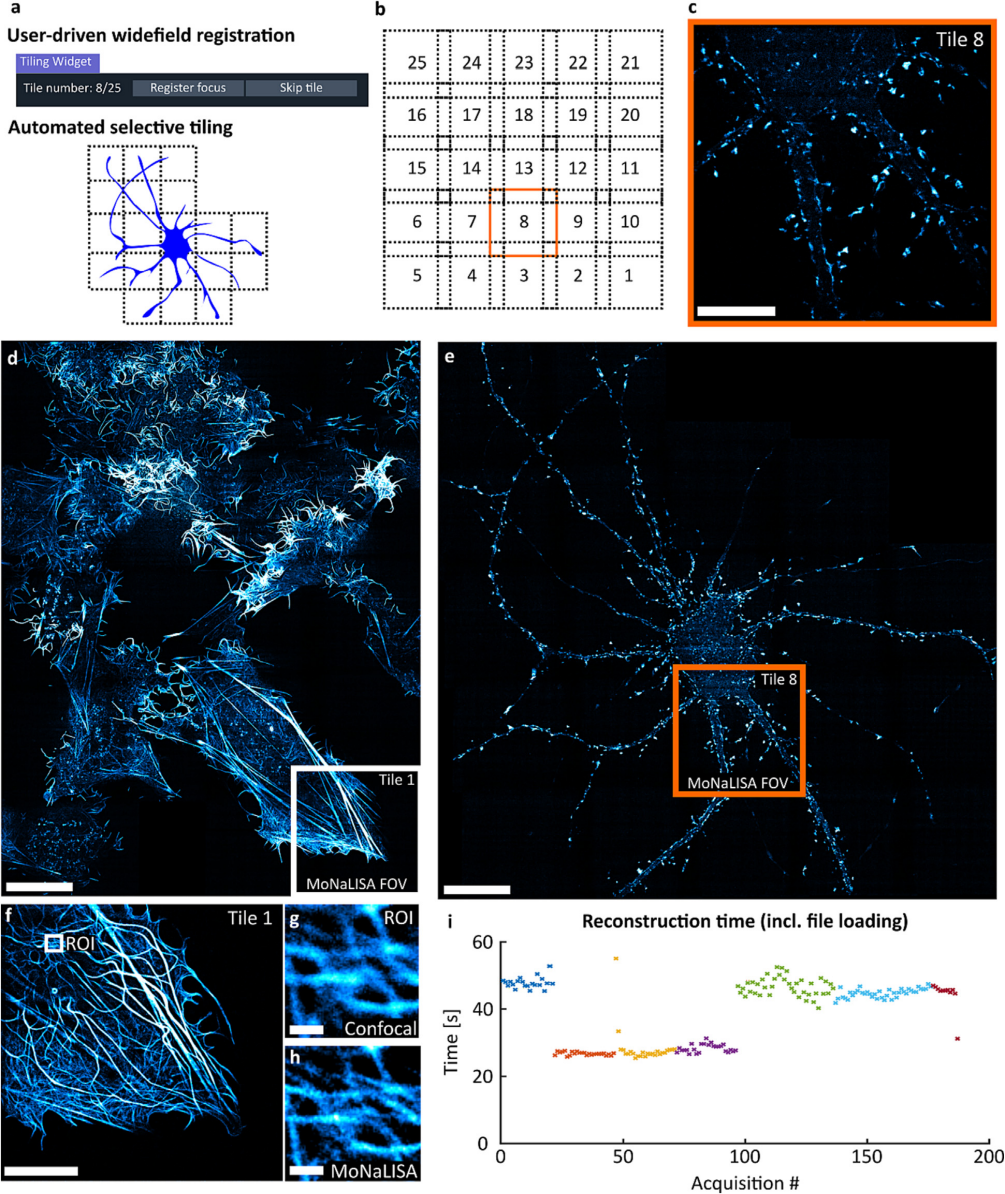
Selective tiling for extending the FOV to 160x160µm^2^ (25x25 tiles). **a** The focus positions are registered by the user using a widefield laser and an ImSwitch widget. The user can skip the tile, thus adapting to the image content. The microscope then performs selective tiling by imaging only the selected areas. **b-c** Schematic of the tiling scheme, including 14 % overlap, and visualization of a single tile (38x38µm^2^). **d-e** Imaging the actin cytoskeleton of U2OS cells and primary hippocampal neurons expressing actinChromobody-rsEGFP2. Scale bar 20 µm. **f-h** zooming in Tile 1 in the U2OS cells (d), and comparison between confocal and MoNaLISA. Scale bars 5 µm and 0.5 µm, respectively. **i** Reconstruction time of 200 executions using a remote instance. Each color represents a different experiment, and each data point is a single image acquisition (or tile). The values were extracted from the logger files for all the experiments performed using the framework (200 acquisitions).

We developed a tiling script that interacts with the user in order to perform selective tiling. The script consists of two steps. Firstly, user-driven registration of focus and tiles is performed, with the possibility of annotating the tiles that can be skipped because they do not contain biological information. Secondly, the microscope performs automatic tiling of the selected tiles, performing a MoNaLISA scan in each position (Fig. 9a). The stage moves automatically between the tiles, and the script interacts with the user through a dedicated widget (*TilingWidget*).

The MoNaLISA optical setup used has a field of view of 38 × 38 µm^2^ and collects the raw data by scanning the sample with piezoelectric actuators (*3-axis NanoMax* stage from Thorlabs). To move in between fields of view, we employed the stepper motors of the stage controlled through USB and integrated into ImSwitch using the *BSC203Manager* (which calls the python library *thorlabs-apt-devices*), and a 14 % overlap between tiles. The raw data consist of a stack of 324 frames and the acquisition time is 8 ms per frame (2.6 s per image), and we added an extra time of 1 s after each stage movement to prevent from drift. Because of the tilt between the sample and the stage, the z position was slightly different for each tile, which is why we added a widefield-based registration. This process could be further improved by characterizing the tilt or automatically finding the focus in each tile. We used the Fiji plugin MosaicJ for stitching the images [56].

We performed 5x5 tiles (Fig. 9b) to extend the FOV from 38 × 38 µm^2^ (Fig. 9c) to 160 × 160 µm^2^ (Fig. 9d-e) while keeping the super-resolution features introduced by the microscope (Fig. 9f-h).

Furthermore, we extracted the reconstruction time of the remote unit from the logger files. The logger files contain information about the processing computer and the time of execution of each file. We displayed the reconstruction time for 200 acquisitions in Fig. 9i. The total reconstruction time ranged between 25 and 45 s when using an external unit and 7–10 s if the unit ran on the same computer as the acquisition machine.

### Evaluation of acquisition and reconstruction latency and resources

The system can be employed either by using multiple computers or running ImSwitch instances within the same machine. In order to evaluate whether multiple computers are necessary, the workload of each process needs to be assessed for each application. In Table 1. we characterize the occupied resources of the acquisition and reconstruction units in terms of RAM, CPU usage, and computation time for the proposed use cases. We have done so by using the Resource Monitor in Windows for the RAM and CPU usage and the *datetime* python library directly in ImSwitch to precisely compute the timings.

**Table 1:** Study of the computation resources needed for acquisition and reconstruction.

	Idle (control)	Acquisition	Reconstruction from disk
RAM (total 64 GB)	6 GB	8.6 GB	15–23 GB
CPU usage	0 %	8–13 %	6 %
Time	–	2 s MoNaLISA image 35–55 MB/s writing time of experimental data.	0.4–0.5 s reconstruction algorithm 7–10 s local unit (incl. file loading) 30–50 s remote unit (incl. file loading)

Results show that CPU usage is always low and close to idle (control) since our experiments use digital triggering from an external data acquisition card. Also, the raw data is directly saved on a hard drive. CPU usage would be higher in the case of microscopy modalities that require software triggering or real-time image processing. In our use cases, the RAM is the limiting factor, occupying > 30 GB in total (especially in the reconstruction unit). Therefore, using a separate computer for the reconstruction unit is beneficial for computers with less available memory. In our proof of concept, the reconstruction time would then increase from 7 to 10 s to 30–50 s. This could be further improved using higher-speed networks or higher-performance devices for computation or optimizing the Zarr data saving (e.g., chunk size).

The reconstruction time is always more extensive than the acquisition time. Therefore, the user performing image reconstruction using the GUI manually would impose an evident bottleneck. This is why, previously, the reconstruction was usually performed after the entire acquisition had finished. By distributing the resources and using multiple units for acquisition and reconstruction, the tasks can be performed simultaneously without compromising the temporal resolution of the microscope.

By characterizing the requirements of each microscope application, this framework could be extended to low-cost computing devices for both acquisition and reconstruction, such as Raspberry Pi and Jetson Nano.

To conclude, we have designed and developed a new framework for simultaneously performing acquisition, reconstruction, and visualization for microscope automation. The solution is implemented using the ImSwitch and napari software packages and is easily generalizable to other software. We validated the performance by applying it in the context of super-resolution microscopy and the distribution of computation resources to achieve higher throughput. We performed tiling and timelapse experiments to observe mitochondria and actin in U2OS cells and actin in primary neurons.

## Ethics statements

Primary cultures of hippocampal neurons were prepared from rat embryos. All procedures were performed in accordance with the animal welfare guidelines of Karolinska Institutet and were approved by Stockholm North Ethical Evaluation Board for Animal Research.

## Funding

This work was supported by the Chan Zuckerberg Initiative (napari Plugin Accelerator program, project “Integrating Technology to Improve Real-Time Microscopy”), the European Union (Horizon 2020 program, project IMAGEOMICS 964016), and Vinnova (2020-04702 Imaging-omics).

## Declaration of Competing Interest

The authors declare that they have no known competing financial interests or personal relationships that could have appeared to influence the work reported in this paper.
